# Long-term outcomes of endoscopic sinus surgery for chronic rhinosinusitis with and without nasal polyps

**DOI:** 10.5935/1808-8694.20130055

**Published:** 2015-10-04

**Authors:** Juliana Gama Mascarenhas, Viviane Maria Guerreiro da Fonseca, Vitor Guo Chen, Caroline Harumi Itamoto, Camila Atallah Pontes da Silva, Luis Carlos Gregório, Eduardo Macoto Kosugi

**Affiliations:** a MD graduated at UFG (Second-year ENT resident physician at UNIFESP-EPM).; b MD, ENT, graduated at UNIFESP-EPM (Pediatric ENT Fellow at UNIFESP-EPM).; c Rhinology Fellow at UNIFESP-EPM (Rhinology Fellow at UNIFESP-EPM).; d Rhinology Fellow at UNIFESP-EPM (Assisting Physician in the Rhinology Service - Skull Base - at UNIFESP-EPM).; e PhD in Medicine at UNIFESP-EPM (Head of the ENT and HNS at UNIFESP-EPM).; f PhD in Sciences at UNIFESP-EPM (Coordinator of the Rhinology Fellowship Program and Head Preceptor of the ENT Residency Program at UNIFESP-EPM). Rhinology Division - Department of Otorhinolaryngology and Head and Neck Surgery UNIFESP - EPM (Paulista School of Medicine - Federal University of São Paulo).

**Keywords:** nasal polyps, natural orifice endoscopic surgery, quality of life, sinusitis, treatment outcome

## Abstract

Chronic rhinosinusitis (CRS) significantly affects patient quality of life. Medical and surgical treatments aim to clinically manage the condition.

**Objective:**

To assess the long-term quality of life and clinical management of CRS in patients submitted to endoscopic sinus surgery.

**Method:**

This prospective cross-sectional cohort study enrolled 38 patients and looked into the follow-up data of subjects diagnosed with CRS before surgery, three months after surgery, and at least two years after surgery. The Sinonasal Outcome Test 22 (SNOT-22) was used to assess response to treatment and long-term clinical management of the disease.

**Results:**

Significant improvements in the SNOT-22 scores were seen between the preoperative (61.3) and postoperative assessments with three (16.9) and 24 (32.3) months. No statistically significant differences were seen when patients with polyps were compared to polyp-free subjects. Few patients were controlled in both groups, and 7.89% of the subjects had revision surgery during the study.

**Conclusion:**

Endoscopic sinus surgery significantly improved the quality of life of patients with chronic rhinosinusitis. Clinical control of the condition was acceptable, with few patients requiring re-operation within two years of the first surgery.

## INTRODUCTION

Chronic rhinosinusitis (CRS) significantly affects the quality of life of patients. Cases of CRS may or not be associated with nasal polyps. CRS treatment aims to attain clinical control of the disease, which is defined as the elimination or mitigation of patient symptoms to a point where subjects are no longer bothered by the disease, possibly in combination with a healthy or quasi healthy mucosa requiring only the administration of topical medication^1^.

The severity of symptoms and the impact of the disease upon patient quality of life can be assessed through the Sinonasal Outcome Test 22 (SNOT-22)^2^, ^3^, ^4^. This validated tool encompasses all major symptoms included in the diagnosis criteria set in the European Position Paper on Rhinosinusitis and Nasal Polyps (EPOS) 2012 for CRS^1^. The SNOT-22 is a repeatable tool and the graphic representation of test results allows for easy visualization of the outcomes of conservative and surgical approaches, as well as exacerbations observed during follow-up^2^, ^3^, ^4^. Morley & Sharp^5^ compared 15 sinonasal questionnaires and concluded that the SNOT-22 is the most adequate tool to analyze patients with CRS, including subjects submitted to functional endoscopic sinus surgery. The SNOT-22 was recommended by the EPOS 2012 as the tool to assess patients with CRS^1^.

This chronic illness is correlated with partially explained complex inflammatory mechanisms and host-environment interactions, which together explain the ineffectiveness of medical and surgical therapies in curing patients^1^. Some authors have looked into quality of life and long-term clinical management of the disease^6^, ^7^, ^8^, ^9^, ^10^, ^11^, ^12^, but few were able to show improvements in quality-of-life test scores^7^, ^8^, ^10^. This study aimed to assess quality of life and long-term clinical management of CRS of patients submitted to endoscopic sinus surgery.

## METHOD

This prospective longitudinal cohort study included chronic rhinosinusitis patients followed up for at least two years after endoscopic sinus surgery. The individuals included in the study were recruited from the institution's clinic. They were 18 and older and had been diagnosed with chronic rhinosinusitis with nasal polyps (CRSwNP) or without nasal polyps (CRSsNP) based on the EPOS 2012 criteria, with indication for surgery. The study was approved by the Research Ethics Committee and granted permit 1135/09. Participants signed an informed consent term.

**Figure 1:**
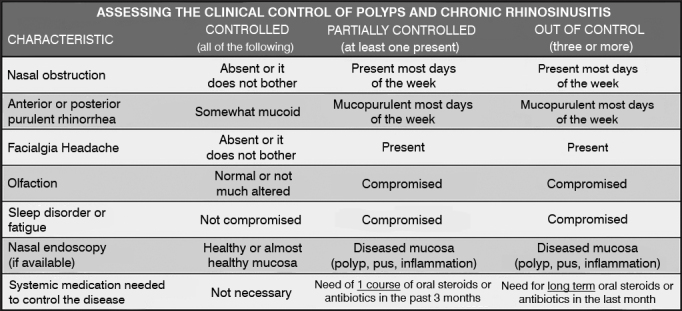
Assessment of chronic rhinosinusitis and nasal polyp medical management. Adapted from EPOS 2012.

Patients were asked to answer the SNOT-22 questionnaire before surgery (Preop) and three months after surgery (PO3m). In the review patients followed up for two years and longer were included (POT). Subjects were given a thorough questionnaire that included the SNOT-22, an assessment of the clinical management of the disease, and reports of revision procedures they were submitted to within the timeframe of the study. The chart proposed in the EPOS 2012^1^ was used to assess the clinical management of CRS (Figure 1).

The Chi-square test (Fisher's exact test or Freeman-Halton extension of Fisher's exact test when needed) was used to analyze the distribution of genders in the CRSwNP and CRSsNP groups. The mean ages of the CRSwNP and CRSsNP groups were compared through the unpaired *t*-test. The mean scores in Preop, PO3m and POT of each group were compared using the paired t-test. The mean scores of the CRSwNP and CRSsNP groups in each of the follow-up phases (Preop, PO3m, POT) were compared through the unpaired *t*-test. The distributions of disease management and management characteristics for each group were compared through the Chi-square test (Fisher's exact test or Freeman-Halton extension of Fisher's exact test when needed). Statistical significance was attributed when *p* < 0.05.

## RESULTS

Sixty patients in preoperative care for endoscopic sinus surgery diagnosed with CRSwNP or CRSsNP were enrolled in the study. After signing informed consent terms, they answered the SNOT-22 questionnaire before surgery and three months after surgery. Thirty-eight of the 60 original patients were found two years after surgery and were included in the second part of the study. Patient characteristics are described on Table 1.

**Table 1:** Participant characteristics.

Characteristics		Groups						Test	*p*-value
		CRSwNP		CRSsNP		Total			
Participants	(N/%)	17	44.74	21	55.26	38	100	-	-
Females	(N/%)	10	58.82	12	57.14	22	57.89	χ^2^	0.92
Males	(N/%)	7	41.18	9	42.86	16	42.11		

Age	(Mean/SD)	50.59	11.39	42.71	14.68	46.24	13.72	*t*	0.07

CRSwNP: Chronic rhinosinusitis with nasal polyps; CRSsNP: Chronic rhinosinusitis without nasal polyps; N: Number; %: Percent in each group; SD: Standard deviation; χ^2^: Chi-square; *t*: Unpaired *t*-test.

**Figure 2:**
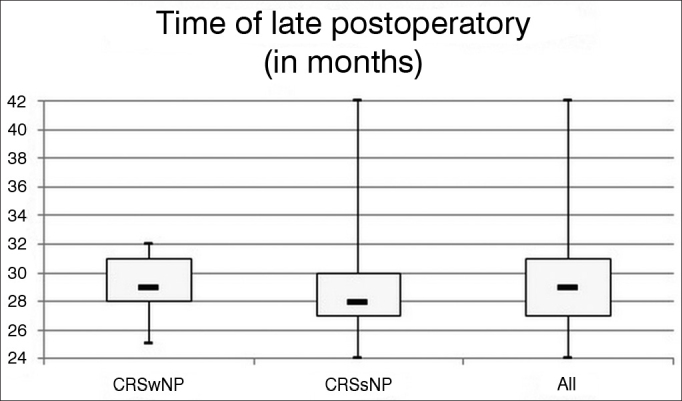
Time distribution of late postoperative follow-up. Minimum time after surgery in late postoperative follow-up was 24 months. No statistically significant differences were seen between the studied groups.

Patients followed up for at least 24 months after surgery were included. There were no statistically significant differences between the CRSwNP and CRSsNP groups in the different follow-up phases (unpaired *t*-test: *p* = 0.72). Mean time after surgery in the late follow-up of CRSwNP subjects was 29.29 months, with a standard deviation of 2.34 months, against 28.95 months and a standard deviation of 3.54 months in the CRSsNP group. The entire sample considered together had a mean POT (late follow-up) of 29.11 months and a standard deviation of 3.03 months. The distribution of POT times can be seen in Figure 2.

The mean values and standard deviations of SNOT-22 scores in Preop, PO3m, and POT are presented in Table 2.

**Table 2:** SNOT-22 scores during follow-up.

	Groups					
	CRSwNP		CRSsNP		Total	
Time	Mean	SD	Mean	SD	Mean	SD
Before surgery	58.88	26.14	63.24	22.71	61.29	24.06
Three months after surgery	14.82	12.58	18.67	16.58	16.95	14.86
Late postoperative follow-up	34.06	24.54	30.95	22.23	32.34	23.02

Paired *t*-test CRSwNP:

Pre vs. PO3m: *p* < 0.0001*; Pre vs. POT: *p* = 0.008*; PO3m vs. POT: *p* = 0.008*

Paired *t*-test CRSsNP:

Pre vs. PO3m: *p* < 0.0001*; Pre vs. POT: *p* = 0.0001*; PO3m vs. POT: *p* = 0.05*

Paired *t*-test combined:

Pre vs. PO3m: *p* < 0.0001*; Pre vs. POT: *p* = 0.0001*; PO3m vs. POT: *p* = 0.001*

Unpaired *t*-test CRSwNP and CRSsNP:

Pre: *p* = 0.59; PO3m: *p* = 0.42; POT: *p* = 0.69

CRSwNP: Chronic rhinosinusitis with nasal polyps; CRSsNP: Chronic rhinosinusitis without nasal polyps; SNOT-22: SinoNasal Outcome Test - 22 questions; SD: Standard deviation; POT: Late postoperative follow-up.

**Figure 3:**
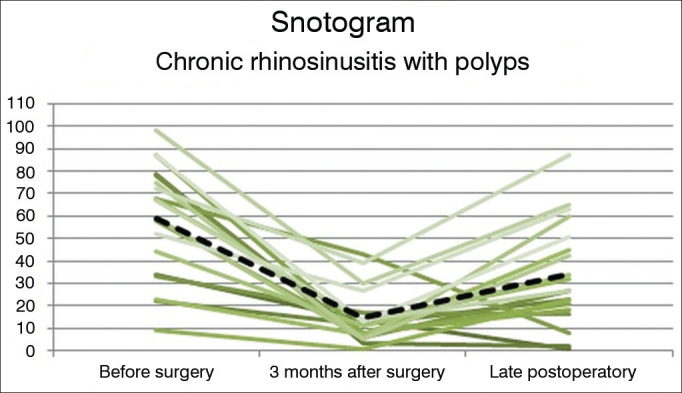
Snotogram of patients with chronic rhinosinusitis with nasal polyps. Dashed line: Mean scores. Marked improvement was seen three months after surgery, along with progressive deterioration of the mean scores in the long term.

**Figure 4:**
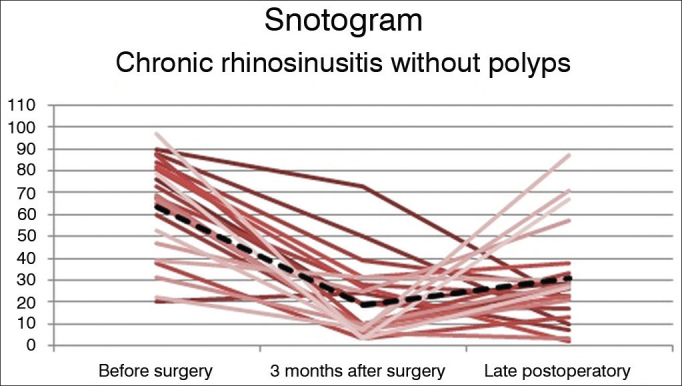
Snotogram of patients with Chronic Rhinosinusitis without Nasal Polyps. Dashed line: Mean scores. Marked improvement was seen three months after surgery, along with progressive deterioration of the mean scores in the long term.

**Figure 5:**
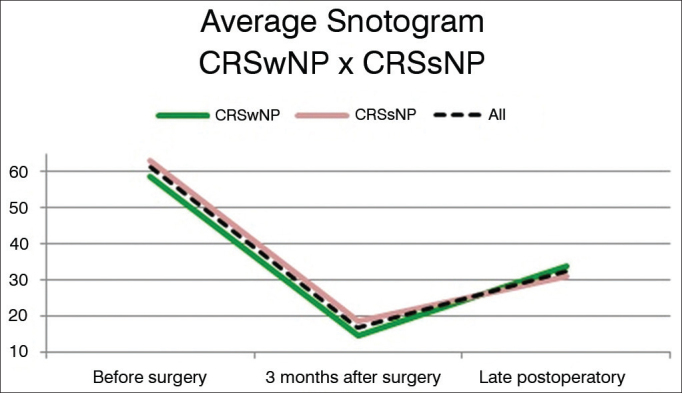
Snotogram of mean scores. Despite the absence of statistically significant differences between groups in each stage of follow-up, the scores of the group with nasal polyps were worse in late postoperative follow-up.

Statistically significant differences were seen between the Preop, PO3m, and POT measurements in all studied groups. No statistically significant differences were seen between CRSwNP and CRSsNP when they were compared in each of the follow-up phases (Preop, PO3m, POT). The before and after surgery measurements are seen in Figure 3, Figure 4, Figure 5 (“Snotograms”).

The clinical management of CRSwNP and CRSsNP patients is shown in Table 3. No statistically significant differences were seen between the groups, but few patients were controlled. Three (7.89%) of the 38 patients underwent revision surgery during follow-up.

**Table 3:** Disease medical management in late postoperative follow-up.

	Groups					
	CRSwNP		CRSsNP		Total	
Characteristic	N	%	N	%	N	%
Controlled	2	11.76	3	14.29	5	13.16
Partially controlled	6	35.29	11	52.38	17	44.74
Uncontrolled	9	52.94	7	33.33	16	42.11
Total	17	100	21	100	38	100

Freeman-Halton extension of Fisher's exact test: *p* = 0.25. CRSwNP: Chronic rhinosinusitis with nasal polyps; CRSsNP: Chronic rhinosinusitis without nasal polyps; N: Number; %: Percent in each group.

The main complains during late follow-up were rhinorrhea (55.26% of the subjects) and hyposmia (50%), as seen in Table 4. No statistically significant differences were seen between the CRSwNP and CRSsNP groups in regards to complaints.

**Table 4:** Characteristics of disease management in late postoperative follow-up.

	Groups						Test
	CRSwNP		CRSsNP		Total		
Characteristic	N	%	N	%	N	%	χ^2^
Obstruction	8	47.06	6	28.57	14	36.84	*p* = 0.24
Rhinorrhea	11	64.71	10	47.62	21	55.26	*p* = 0.29
Facial pain	6	35.29	8	38.10	14	36.84	*p* = 0.86
Hyposmia	10	58.82	9	42.86	19	50.00	*p* = 0.33
Disordered sleep	7	41.18	8	38.10	15	39.47	*p* = 0.84
Systemic medication	6	35.29	6	28.57	12	31.58	*p* = 0.64
Total	17	100	21	100	38	100	-

CRSwNP: Chronic rhinosinusitis with nasal polyps; CRSsNP: Chronic rhinosinusitis without nasal polyps; N: Number; %: Percent in each group; χ^2^: Chi-square.

## DISCUSSION

Endoscopic sinus surgery improved the quality of life of the patients with chronic rhinosinusitis enrolled in this study. Despite the worse quality-of-life scores in early postoperative follow-up (three months) when compared to late postoperative care (24 months and longer), the SNOT-22 and POT scores were statistically better than before surgery, indicating the improvement in quality of life produced by surgery was sustained in the long term. Such late postoperative improvement in quality of life contradicted other studies in which SNOT-22^7^ or other tests^8^, ^9^, ^10^, ^11^ were used. Even when different parameters were used to measure postoperative outcomes, the trends indicated that the disease would maintain a stable course in most cases^6^.

Hopkins et al.^7^ found mean scores of 42.0 in the SNOT-22 before surgery, and statistically significant improvements to 25.5 in early postoperative and 27.7 in late postoperative follow-up. The scores in postoperative care were not statistically different from each other. The mean SNOT-22 scores in Preop, PO3m, and POT seen in our study were 61.3, 16.9, and 32.3, respectively. The sharper drop seen in the preoperative to recent postoperative follow-up period may have accounted for the statistically significant difference seen between early and late postoperative follow-up scores, as late follow-up scores in this study were similar to the scores reported by Hopkins et al. Statistically significant improvements in quality of life were observed two years after surgery, in addition to gradual deterioration after three months of surgery, although not statistically significant.

Considering the late follow-up clinical management of CRS, more than half of the patients were managed in acceptable levels (13.2% controlled and 44.7% partially controlled). However, 42.1% of the patients were characterized as having their condition uncontrolled, stressing the chronic character of CRS. Despite the improvements in quality of life, patients are not free of disease and go through periods of exacerbation^1^. The rate of subjects with controlled disease found in this study was below expected levels. Despite the lack of data on disease clinical management based on the EPOS 2012^1^ criteria, Rowe-Jones et al.^6^ reported a success rate of clinical management of 89% five years after surgery. The authors defined failure as the need for at least one course of salvage medication per month in two consecutive months. They also found that 36% of the patients required additional treatment with steroids and antibiotics throughout the five years of postoperative follow-up. Li et al.^13^ used clinical history parameters and endoscopic findings to define clinical control, and reported a rate of success of 87% within 24 months. Hopkins et al.^7^ found that 50% of the patients took medication for sinus disease throughout the follow-up period. Published revision surgery rates range between 4.2% and 11% within 36 to 60 months of follow-up^6^, ^7^; the 7.9% found in our study was within that range, despite the shorter duration of the study.

Rhinorrhea (55.3%) and hyposmia (50%) where the main complaints present in late follow-up. Nasal obstruction is the most prevalent complaint at the time of diagnosis^1^, and is reported by 96.5% of patients with CRSwNP and 93.5% of subjects with CRSsNP^14^. Nonetheless, patients usually improve from this symptom after surgery^3^, ^9^, ^15^. Posterior rhinorrhea (87.4%)^9^ and olfactory/ gustatory alterations (90.3% in CRSwNP; 75.7% in CRSsNP)^14^ are next, as also seen in the postoperative data gathered in our study, considering that nasal obstruction improves to a greater degree than other symptoms. Damm et al.^9^ observed improvements of 84% in nasal obstruction, 77.8% in rhinorrhea, and 73.2% in hyposmia after sinus surgery, as also reported in the review by Chester et al.^15^, in which moderate improvements in facial pain and posterior rhinorrhea and minor improvements in hyposmia and headache were described. In view of these findings, the prevalences described in our study are within the expected range for patients in postoperative follow-up.

Nasal endoscopy and imaging were not used in this study to quantify the clinical management of chronic rhinosinusitis. After all, chronic rhinosinusitis is a symptom-based condition, and symptom-free patients usually do not require additional treatment, even when they present minor alterations in endoscopic examination or mucosal thickening in CT scans of the paranasal sinuses^16^. For that reason, subjective improvement scores tend to be higher than their objective counterparts derived from endoscopic examination, mucociliary clearance, odor detection thresholds, and nasal volume assessment^6^. Nonetheless, nasal endoscopy is routinely used in preoperative visits to adequately assess and manage patients with chronic rhinosinusitis, and CT scans of the paranasal sinuses are reserved for cases of treatment failure and patients with multiple symptoms despite adequate postoperative medical management^16^.

Many authors have reported acetylsalicylic acid intolerance^10^, ^11^, ^17^, asthma, depression^11^, previous sinus surgery^11^, ^13^, peripheral eosinophilia absolute counts above 520/µL, eosinophilia in the mucosa or mucus^17^, allergy^12^, ^13^, CRSwNP^13^ and previous polypectomy^12^ as predictors of poor response to surgery for CRS. Li et al.^13^ observed a success rate of only 52.3% in the treatment of patients with recurrent nasal polyps. The authors advocated first line therapy with systemic or topical steroids for these patients. Matsuwaki et al.^17^ showed that eosinophilic CRS was strongly correlated with recurrence in five years and recommended examination of the inflammatory infiltrate of the nasal polyps or the paranasal mucosa and long-term administration of anti-inflammatory drugs after surgery.

Despite the improvements in symptoms and quality of life provided by sinus surgery, it fails to act on certain aspects of the complex pathophysiology of chronic rhinosinusitis and cannot cure patients alone. Long term follow-up is of the essence, but proper management can be challenging given the significant demand faced by large public health care systems and the difficulty performing rigorous clinical reviews. The deterioration in clinical management and increase on SNOT-22 scores seen two years after surgery probably reflect the combination of the disease's natural history and the lack of timely administration of complementary drug therapy.

## CONCLUSION

Endoscopic sinus surgery significantly improved the quality of life of patients with chronic rhinosinusitis. Patients had acceptable levels of medical management of their condition and few required re-operation within two years of surgery.
